# Challenges associated with limited‐resources cancer care facility: Bilateral breast myeloid sarcoma as an example

**DOI:** 10.1002/ccr3.2407

**Published:** 2019-09-16

**Authors:** Najmaddin Khoshnaw, Ahmed K. Yassin, Alaa F. Alwan, Hemin A. Hassan, Layth Mula‐Hussain

**Affiliations:** ^1^ Department of Hematology Hiwa Cancer Hospital Sulaymaniyah Kurdistan Iraq; ^2^ Program of Hematology Kurdistan Board of Medical Specialties Erbil Kurdistan Iraq; ^3^ Department of Medicine College of Medicine Hawler Medical University Erbil Kurdistan Iraq; ^4^ Department of Clinical Hematology National Center of Hematology Mustansiriyah University Baghdad Baghdad Iraq; ^5^ Department of Hematopathology Shorash Hospital Sulaymaniyah Kurdistan Iraq; ^6^ Division of Radiation Oncology Zhianawa Cancer Center Sulaymaniyah Kurdistan Iraq; ^7^ Program of Radiation Oncology Kurdistan Board of Medical Specialties Erbil Kurdistan Iraq

**Keywords:** breast mass, hematology, Iraq, limited resources, myeloid sarcoma, oncology

## Abstract

Myeloid sarcoma (MS) is a rare myeloid malignancy. It can arise before, concurrent with, or following different malignant hematological diseases, most commonly acute myeloid leukemia (AML), myelodysplastic syndrome, or myeloproliferative neoplasms. Here we describe a 30‐year‐old female with AML‐M1 who presented to the hematology department with bilateral breast pain and tenderness. Available diagnostic measures including ultrasound of breast and magnetic resonance imaging were used to determine the nature of these breast lumps. MS was definitively diagnosed via biopsy and the patient was treated with systemic chemotherapy. Despite her AML treatment she unfortunately died secondary to disease progression. The authors consider this case of particular educational value due to the bilateral and aggressive nature of this patient’s disease in the setting of a cancer‐care facility with limited resources.

**Key Clinical Message:**

In retrospect, if breast MS had been considered earlier in this patient’s presentation, a referral to an outside center with matched stem cell transplantation capability may have been warranted after complete remission following first bone marrow relapse, rather than continuing chemotherapy alone.

## Abstract 2

In retrospect, if breast myeloid sarcoma had been considered earlier in this patient's presentation, a referral to an outside center with matched stem cell transplantation capability may have been warranted after complete remission following first bone marrow relapse, rather than continuing chemotherapy alone.

## Graphical abstract

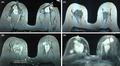

## 1 OVERVIEW

The extramedullary presentation of acute leukemia is a known manifestation that can occur in a variety of organs or tissues. MS is one of these manifestations.^1^ MS, also known as granulocytic sarcoma, refers to the infiltration of extramedullary parts of the body by granulocytic or monocytic neoplastic precursors.^2^ Many risk factors such as cellular immune dysfunction, malnutrition, high leukocyte count, myeloblasts with T‐cell markers, and genetic/chromosomal changes have been described. According to the WHO classification (2016) of hematological malignancies, MS is considered a subtype of AML and should be viewed as an equivalent to AML or considered as blast transformation when it occurs in the context of a myeloproliferative or myelodysplastic syndrome.^3^, ^4^, ^5^, ^6^, ^7^, ^8^

Myeloid sarcoma has been reported in 2%‐8% of patients with AML, either as single or multimodal tumors. In about 25% of the cases, it precedes AML,^9^ while it appears concomitantly in 15%‐35% and occurs after the diagnosis of AML in 50% of cases. MS may also occur as an initial manifestation of relapse in previously treated AML.^10^, ^11^, ^12^ In all these cases, the age of distribution of this pathology is extremely variable, ranging between 1 and 81 years.^13^

The diagnostic challenge for MS is when it presents as an isolated or primary disorder. The absence of leukemic cells in the bone marrow makes MS easily confused with other hematological malignancies, such as lymphoma or even nonhematological malignancies such as melanoma or Ewing's sarcoma.^14^, ^15^

Given the wide variety of anatomical sites which this entity can develop, imaging can facilitate diagnosis and therapeutic monitoring. The imaging method of choice in the identification of these soft tissue masses is most commonly computed tomography (CT). Positron emission tomography (PET) has high sensitivity in the detection of MS in its early stages. Studies indicate that the combined use of CT/PET in the diagnosis of MS has greater specificity and sensitivity than the isolated use of either method.^16^, ^17^ Recently, reported data have determined that MRI is superior to more conventional screening techniques, particularly in those with the previous history of breast cancer.^18^

For early diagnosis of MS cooperation and collaboration between haematologists and pathologists is key as well as the use of different immune‐histochemical (IHC) and cytogenetic studies. However, currently specific markers for this pathology are sparsely described. The most commonly used markers in the diagnosis of this entity are MPO, CD68, and CD43, which represent high sensitivity but low specificity. To maximize diagnostic accuracy, most studies also suggest the use of CD33, CD34, and CD117. Cytogenetic abnormalities, which may occur in MS, are similar to those seen in AML with t (8.21) and inv 16, most frequently reported.^19^

Although the therapeutic modalities for MS are not fully defined, there is a consensus that early diagnosis and treatment before the transformation of isolated MS to AML are prerequisites for achieving good clinical outcomes.^19^ In the vast majority of patients, the therapeutic agents of choice should be based on the induction chemotherapy regimen classically used in AML.^19^, ^20^ If solitary MS is not rapidly treated; it can progress to AML within 10 to 12 months.^13^ In most cases, the treatment outcomes are relatively poor; however, they appear to be superior to those obtained in the treatment of AML without the extramedullary disease.^19^, ^20^, ^21^, ^22^, ^23^ The prognosis depends on the primary site of involvement, underline primary disease, and time of presentation (before, during, and after primary disease diagnosis).^24^

The presentation of this clinical case aims to demonstrate the challenges underlying the diagnosis and treatment of bilateral breast MS and provide a new perspective on its prognosis in the context of a rare presentation.

## 2 CASE PRESENTATION

A 30‐year‐old woman with known AML (M1) diagnosed during her first trimester of pregnancy presented to us status postinduction, consolidation, and high‐dose Ara‐C chemotherapy. Four months after her last treatment, she relapsed in the bone marrow and received two courses of FLAG‐IDA chemotherapy and bone marrow remission was achieved. Unfortunately, due to unavailability of a compatible donor, she could not be offered bone marrow transplantation.

**Figure 1:**
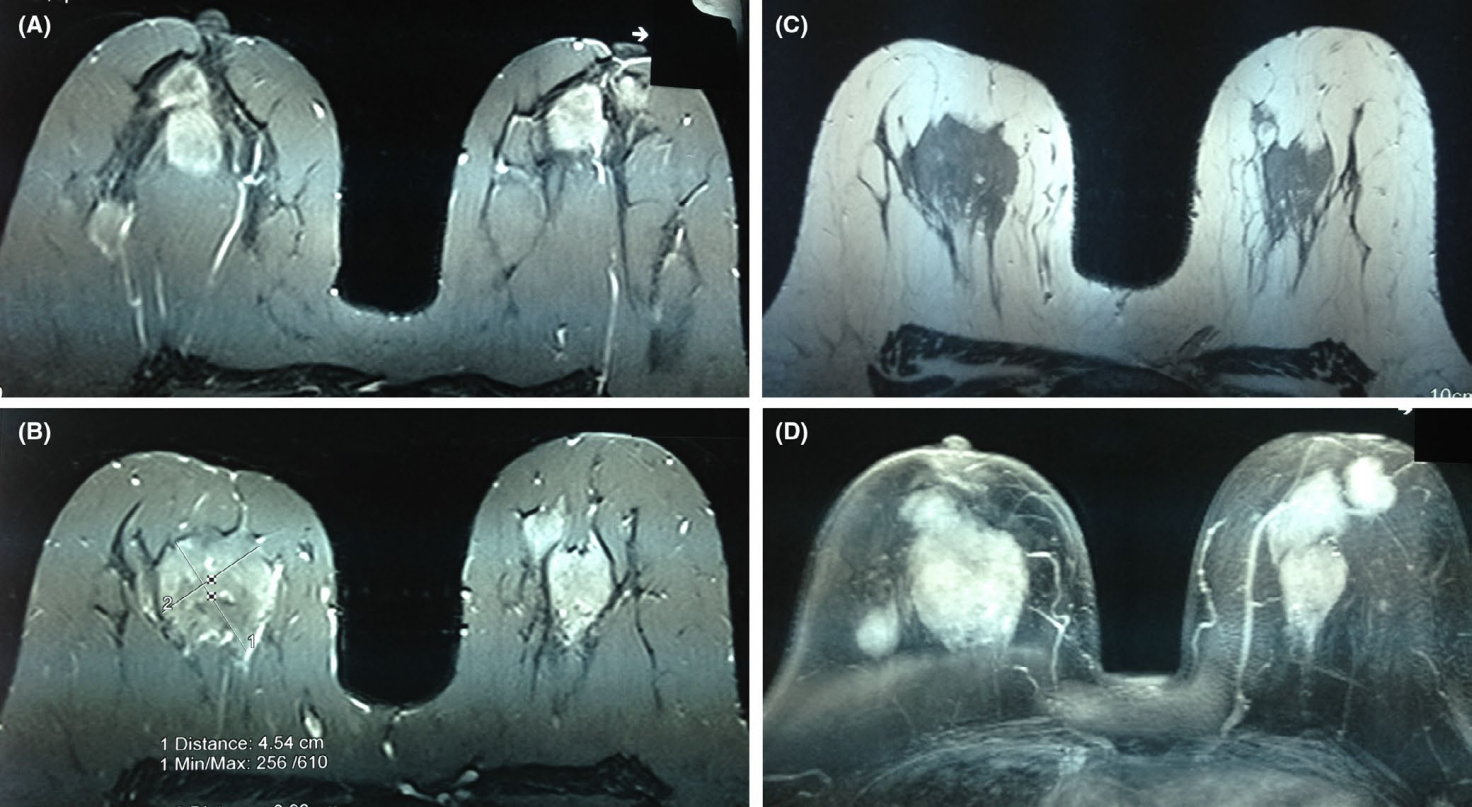
A‐D, Breast MRI, with bilateral multiple masses, two in the right and three in the left. The largest was in the right breast, measuring 45 × 40 mm (A, B). The masses were hypo‐intensive in T1 (C) and hyper‐intensive in T2 (D)

**Figure 2:**
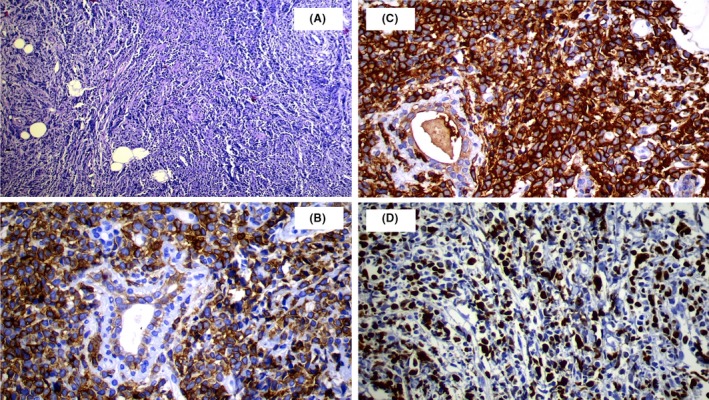
A‐D, Blastic cells, without late differentiation (A). IHC is positive for CD 117 (cytoplasmic membrane staining), CD45, Ki67 (B‐D)

**Figure 3:**
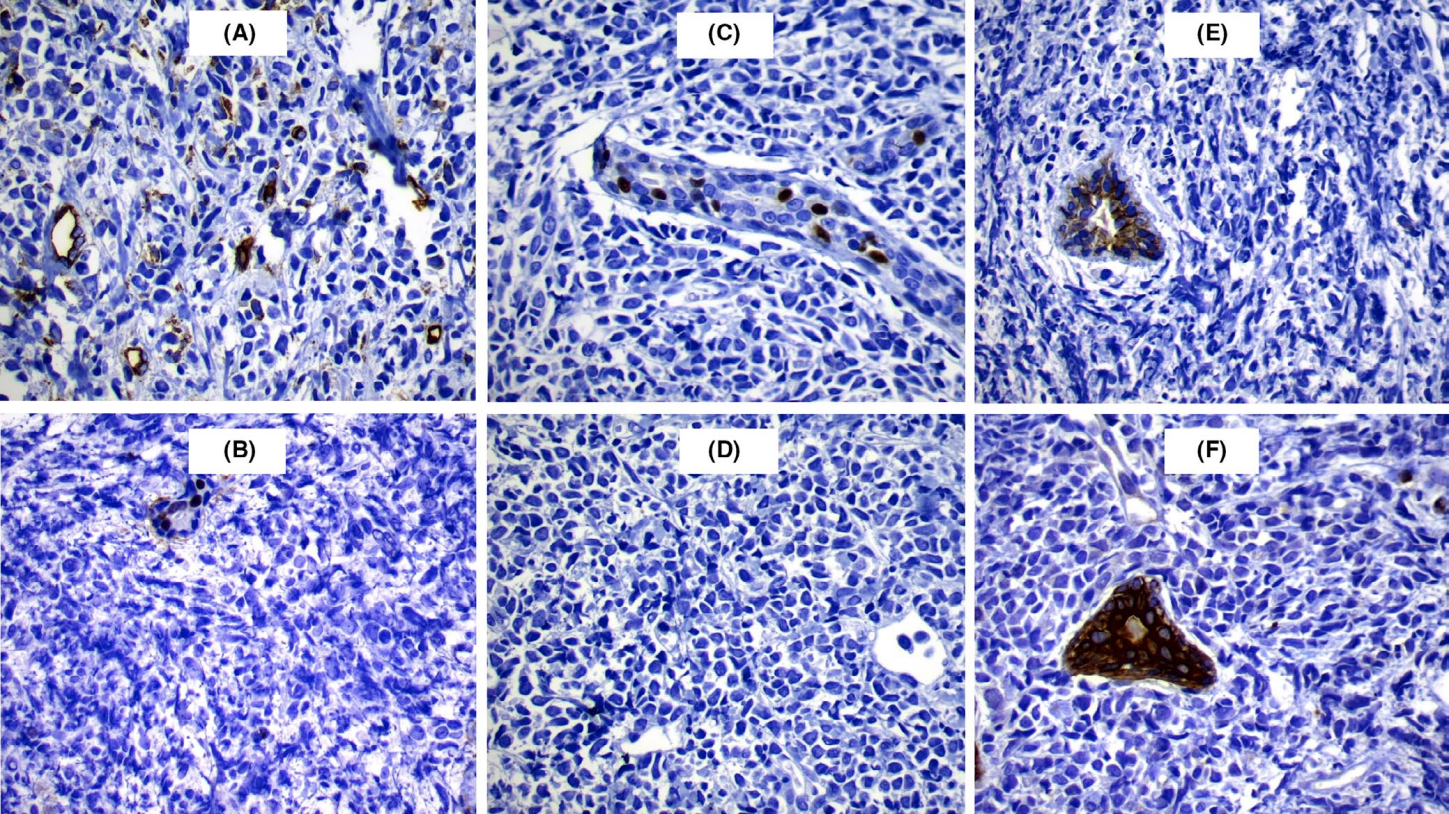
A‐F, Cells are negative for CD34, ER, PR, HER2, E‐cadherin, AE1 and AE3

She subsequently presented to our hematology department with bilateral breast pain and tenderness of two weeks duration. Her symptoms started eight weeks after demonstration of bone marrow remission. Sonography showed oval‐shaped, heterogeneous, echogenic bilateral masses, 41 × 26 mm and 22 × 14 mm, in the right and left breasts, respectively, with normal vascularity. Breast MRI showed multiple masses in each beast, round‐shaped with a mild, irregular outline, two in the right and three in the left breast. The largest lesion was in the right breast, measuring 45 × 40 mm (Figure 1A‐B). The masses were hypo‐intensive in T1 (Figure 1C) and hyper‐intensive in T2 (Figure 1D). Core needle biopsies revealed diffuse infiltration by medium‐sized atypical cells with moderate cytoplasm and vesicular to folded nucleoli. Cells showed frequent mitotic figures and distinct perivascular arrangement. The cells were blastic, without late differentiation (Figure 2A). Her IHC was positive for CD 117 (cytoplasmic membrane staining), CD45, Ki67 (Figure 2B‐D), and negative for CD34, ER, PR, HER2, E‐cadherin, AE1 and AE3 (Figure 3A‐F). A diagnosis of MS was determined, and the patient received FLAG‐IDA chemotherapy protocol. Unfortunately, the patient did not obtain complete remission and subsequently perished secondary to disease progression.

## 3 DISCUSSION

Myeloid sarcoma usually occurs in different organs and exhibits rapid growth. MS of the breast is uncommon and may be misdiagnosed as lymphoma or carcinoma, especially if there is no bone marrow involvement. A study by Viadana et al reported only four cases (1.7%) of breast involvement among 235 patients with AML.^25^ MS has been reported in all age groups; however, it occurs most commonly in young adults with a mean age of 37.3 years (range of 12‐79 years). This patient was 30 years old with bilateral breast masses. The most common presentation of patients with MS of the breast is a painless mass, typically not associated with nipple discharge or retraction.^26^ In this case, the patient presented with painful breasts and tenderness.

Imaging studies are minimally helpful in differentiating MS from other malignancies. Mammographic studies often show both regular and irregular edges with increased stromal density and not associated with microcalcifications. Ultrasonography studies of MS may show homogeneously hypoechoic areas with well or poorly defined margins. MRI and CT demonstrate nonspecific heterogeneous densities. In this case, ultrasound showed an oval‐shaped heterogeneous echogenic mass in the right (41 × 26 mm) and left breast (22 × 14 mm) with normal vascularity. MRI showed multiple masses in each beast with irregular shape and outline. As seen in this instance, variable appearance of different imaging techniques to distinguish MS from mammary carcinoma or metastatic tumors can be quite difficult.^27^, ^28^

The definitive histological diagnosis in this case was based on the findings of characteristics of immature myeloid cells in sarcoma with relevant CD markers by IHC stains. Despite its characteristic histological appearance, there is still a risk for diagnostic error for breast MS due to its rarity. The condition may be misdiagnosed as a benign tumor or primary carcinoma of the breast by FNA.^20^ Many patients with isolated MS are mistakenly diagnosed, more often mistaken for non‐Hodgkin's lymphoma, lymphoblastic leukemia, melanoma, Ewing's sarcoma, and extramedullary hematopoiesis.^1^, ^2^ Given that MS often displays positivity to markers of T cells (CD43, CD45 or CD3) and more rarely for B cell markers (CD79a), distinguishing between MS and lymphoma becomes quite challenging. It is recommended to use an IHC panel that includes the aforementioned markers as well as CD33, CD34, CD117, CD3, and CD20.^29^ This panel may include other markers in case other entities are included in the differential diagnosis.^14^ In this case, IHC was positive for CD117 and CD45.

The treatment strategies for MS of the breast remain controversial. The majority of studies have concluded that all patients with MS should undergo mastectomy or lumpectomy with standard systemic chemotherapy.^29^, ^30^, ^31^, ^32^ The above case did not include surgery and unfortunately systemic chemotherapy was not successful, with the patient dying shortly after treatment.

The diagnosis of breast MS should be taken into consideration in patients presenting with breast lumps and AML. Despite its rarity and diagnostic challenge, early treatment with lumpectomy and systemic chemotherapy or stem cell transplantation for MS may be life‐saving.

## CONFLICT OF INTEREST

No conflict of interest by the authors needs to be disclosed.

## AUTHOR CONTRIBUTIONS

The first author drafted the report and the coauthors further refined it and shared in the final shape of the report.
